# Understanding the microbiome in autologous haemopoietic stem cell transplant for multiple sclerosis

**DOI:** 10.3389/fimmu.2025.1590601

**Published:** 2025-06-30

**Authors:** Jun Yin, Nadeem O. Kaakoush, Jennifer Massey, Mark Danta

**Affiliations:** ^1^ UNSW School of Clinical Medicine, University of New South Wales, Sydney, NSW, Australia; ^2^ School of Medical Sciences, University of New South Wales, Sydney, NSW, Australia; ^3^ Blood Stem Cell and Cancer Research Group, St. Vincent’s Centre for Applied Medical Research, Sydney, NSW, Australia; ^4^ Department of Neurology, St. Vincent’s Hospital, Sydney, NSW, Australia; ^5^ Department of Gastroenterology and Hepatology, St. Vincent’s Hospital, Sydney, NSW, Australia

**Keywords:** multiple sclerosis, gut microbiome, autologous haemopoietic stem cell transplant (AHSCT), dysbiosis, immune reconstitution (IR)

## Abstract

**Background:**

MS is a chronic inflammatory and degenerative disease of the central nervous system (CNS) resulting in neurological deficits associated with physical and/or cognitive disability. The gut microbiome can interact with the CNS and immune system through various molecular pathways and has been previously implicated in MS. Autologous Haematopoietic Stem Cell Transplant (AHSCT) in MS arrests inflammatory disease and has evidence of long-term therapeutic benefit. To date, no study has explored the effect of AHSCT on the gut microbiome in people with MS.

**Method:**

The microbiome of people with MS (pwMS) undergoing AHSCT was compared with pwMS on Natalizumab (NTZ). Longitudinal microbiome analysis was also conducted within the AHSCT cohort at two timepoints. Amplicon sequencing of the 16S ribosomal RNA V3–4 region (Illumina MiSeq) was used to evaluate alpha and beta diversity, oral-stool microbiota distances, and relative taxa abundances on both oral and stool microbiota.

**Results:**

The pre-transplant, baseline samples from the AHSCT cohort (n=8) was compared to the Natalizumab group (n=22). The AHSCT cohort had lower oral species richness compared to the NTZ cohort (p=0.026). There was a significant difference in oral beta diversity between the two cohorts (p=0.043). The oral taxa analysis of AHSCT subjects showed increased relative abundances of Porphyromonas and decreased Veillonella.

**Conclusion:**

This pilot study identified specific microbiome changes, particularly in the oral alpha diversity and abundance of specific bacteria which may reflect treatment status or disease activity in MS.

## 1 Introduction

Multiple sclerosis (MS) is a chronic, immune-mediated disease of the central nervous system (CNS) which affects an estimated 2.8 million people worldwide (1). With the average age of diagnosis ranging from 30–40 years old (2), MS causes significant physical and cognitive disability (3).

Genes within the human leukocyte antigen (HLA) complex remain the strongest genetic risk factor for MS (4). However, other epidemiologic risk factors include exposure to Epstein Barr virus, vitamin D levels, geographical location such as latitude, obesity in adolescence and smoking (5). Intestinal microbiota has also emerged as a potential factor influencing MS pathogenesis (6). Activation of an abnormal adaptive immune response is central to disease initiation (7). Self-reactive T and B lymphocytes infiltrate the CNS, crossing the blood-brain barrier (BBB) and triggering inflammatory cascades that result in glial and neuronal injury (8).

Natalizumab (NTZ), is a monoclonal antibody which inhibits T-cell migration into the CNS via α4-integrin inhibition (9). However, its action is non-specific, also disrupting T-cell circulation within the gut (10). This makes NTZ an intriguing drug for studying the gut-immune axis and its potential alternate pathways of action in MS. Autologous hematopoietic stem cell transplant (AHSCT) has demonstrated the potential for immune reconstitution, particularly in patients with more severe, DMT-refractory MS (11). AHSCT involves high-dose chemotherapy to eradicate autoreactive immune cells, followed by the infusion of autologous stem cells to rebuild a novel immune system (7).

The gut microbiome is increasingly recognized as a key player in modulating systemic immunity and CNS inflammation. Microbial metabolites, such as short-chain fatty acids (SCFAs) and neurotransmitters, influence peripheral immune function, while cytokines and chemokines derived from gut bacteria can disrupt CNS homeostasis (12). Conversely, CNS-derived biochemical changes can alter gut microbial composition through the hypothalamic-pituitary-adrenal (HPA) axis (13). Specific gut bacteria, such as segmented filamentous bacteria (SFB), are known to drive T-helper 17 cell differentiation, which is implicated in autoimmune diseases, including MS (14). Dysbiosis, an imbalance in microbial composition or metabolites, can exacerbate neuroinflammation by disrupting intestinal and systemic immune homeostasis (15). Although numerous studies have identified differences in stool microbial taxa between people with MS (pwMS) and healthy controls (HC) (16–21), these findings are largely correlative. It remains unclear whether dysbiosis contributes to MS pathogenesis or is secondary to the disease and its treatments. There are also limited studies characterising the oral microbiome in pwMS. Furthermore, while research on gut microbiota’s role in immune reconstitution following hematopoietic stem cell transplantation (HSCT) in haematological malignancies has revealed intriguing associations, similar studies in pwMS have yet to be conducted.

This present study aims to firstly characterise the differences in the oral and stool microbiome of people with MS in remission on treatment (NTZ) compared with those pre-AHSCT, refractory to available DMT. Secondly, for pwMS undergoing AHSCT, analysis of longitudinal changes in oral and stool microbiota could help identify microbial shifts associated with disease activity or treatment effects, and explore their potential relevance to immune reconstitution.

## 2 Method

### 2.1 Study outline

An observational cohort study was conducted by comparing the oral and stool microbiome of pre-AHSCT pwMS with a control group of pwMS undergoing treatment with NTZ (St. Vincent’s Hospital, Sydney HREC 2021/ETH11173). This allowed for a comparison of the microbiome in active DMT-refractory MS and stable, treated MS. Within the AHSCT cohort, a longitudinal analysis of the oral and stool microbiome was then conducted throughout treatment. All patients provided written consent prior to sample collection.

### 2.2 Cohorts

NTZ Control Cohort: Patients, aged 18-65, with MS as determined by a neurologist according to the 2017 revised McDonald’s criteria (22) were suitable for inclusion in the control cohort. Recruited patients had been receiving NTZ for 3 or more months, 300mg of intravenous NTZ every 4–6 weeks. Pre- and post-AHSCT samples were collected from these patients.

AHSCT Cohort: All patients undergoing AHSCT as part of the AHSCT in Multiple Sclerosis (AIMS) study were required to contribute stool and saliva samples as part of the interventional clinical trial. Participants were off immunotherapy at clinically appropriate wash-out periods prior to the collection of baseline, pre-ASHCT samples (**Appendix 1**). This allowed for a snapshot of the gut microbiome in ‘active’, DMT-refractory MS. See **Table 1**. for the inclusion and exclusion criteria of this cohort.

**Table 1:** Inclusion and exclusion criteria of AHSCT cohort.

Inclusion Criteria
• Diagnosis of relapsing MS made by a neurologist according to the 2017 revised McDonald’s criteria (22). • EDSS score 0 – 6.5 • Active MS despite the use of high efficacy disease modifying therapy* for >6 months prior to the relapse. ‘Active MS’ defined as: o ≥1 clinical relapse in the opinion of the referring neurologist AND/OR o Evidence of radiological disease activity (T1 lesion, T2/FLAIR lesion, Gd+ lesion) and evidence that this new activity did not preclude commencement of high-efficacy DMT. *High efficacy DMT currently includes: natalizumab, ocrelizumab, ofatumumab, alemtuzumab, fingolimod and cladribine.
Exclusion Criteria
• Any patient on the study treatment arm deemed not suitable for transplant by a consensus of a HSCT multidisciplinary team composed of specialist doctors. • Any patient unable to understand the risks and purpose of the study or adhere to the post-transplant management including medication adherence and appointment attendance. • Patients with a predominately progressive form of MS (‘primary’ or ‘inactive secondary’ progressive MS). • Patients where MS mimics have not been adequately excluded. • Patients unable to undergo MRI scans. • Patients with advanced disease where the risks of transplant are deemed to outweigh potential benefits

### 2.3 Patient recruitment

Patients were recruited between 1 Jan 2022 and 1 July 2024. Each patient was assigned a unique study ID, with relevant clinical and demographic data recorded in the institutional REDCap database.

### 2.4 Sample collection and storage

INVITEK Molecular kits were used to collect faecal samples and DNAGenotek kits were used to collect oral samples. For AHSCT patients, sample collection was scheduled before AHSCT treatment (baseline samples), and 6 months post-AHSCT. AHSCT participants were provided with self-collection kits at the 6-month post-AHSCT appointment. However, as samples were self-collected at home and returned via post, actual collection times ranged from 7 to 12 months. Samples were aliquoted and stored at -80°C, which is regarded as the gold standard (23).

### 2.5 Nucleic acid extraction

DNA extraction was conducted at the Microbiome Research Centre, Kogarah, NSW. Frozen stool samples were processed using the INVITEK™ PSP DNA extraction kit. Frozen oral samples were processed using the Qiagen™ DSP pathogen DNA extraction kit.

### 2.6 Sequencing and data analysis

The 16S rRNA V4 regions were amplified by PCR and sequenced on the MiSeq platform (Illumina) using the 2×300 bp paired-end protocol. Sequencing was performed at the UNSW Ramaciotti Centre for Genomics. Raw sequencing data was analysed using Mothur v1.48.1 and vSearch v2.22.1 on the UNSW Katana server (24). The Ribosomal Database Project (RDP) v19 was used as the reference database for taxonomic classification. Operational taxonomic units (OTUs) were used to group clusters of uncultivable microorganisms with sequence similarity as an analytical unit. The numbers represented the degree of importance; the lower the number the more prevalent the genus (i.e. OTU05 is more prevalent than OTU26). Similarity was detected by the 16S taxonomic marker gene and was used to classify microbial individuals at different taxonomic levels.

Primer-E v6 was used to calculate alpha diversity measures including Margalef’s species richness, Pielou’s evenness and Shannon’s diversity index. For both stool and oral samples, the Shaprio-Wilk test was used to test for normality of data. Alpha diversity of normally distributed data was analysed with unpaired t-tests while data for which didn’t fit a normal distribution, Mann-Whitney U tests were used. Beta diversity was calculated with Bray-Curtis dissimilarities from OUT relative abundances (%) that were transformed by square root. Principal-coordinate analysis (PCoA) and permutational multivariate analysis of variance (PERMANOVA) was then calculated. The comparison between the oral and stool microbiota was performed with a PCoA and PERMANOVA on a Bray-Curtis resemblance matrix, and the distance relationship between the oral and stool microbiota was calculated with a Mann-Whitney U test. GraphPad Prism v9.4 was used for statistical analysis.

For taxonomic classification, per taxon analysis was performed using ZicoSeq (25). Counts were log-transformed and only taxa that contributed to at least 1% to the oral and stool microbiota were included. An unpaired t-test was applied and p-values were corrected for false discovery rates (FDR) using the Benjamini- Hochberg method (BH) to form a q-value.

## 3 Results

### 3.1 Subject characteristics

A total of 30 participants with MS were enrolled into the study: 22 in the NTZ cohort and 8 in the AHSCT cohort. The two cohorts were well-matched in terms of sex and age. The AHSCT cohort had a higher Expanded Disability Status Scale (EDSS) median score and higher 12-month antibiotic exposure. No patients in the AHSCT cohort received NTZ within 6-months of treatment. Demographic information about the cohorts is summarised in **Table 2**. Baseline oral and stool samples were compared from all patients in the AHSCT and the NTZ control cohorts. Of the 8 AHSCT patients who provided baseline oral and stool samples (T1 samples), 6 provided longitudinal samples at a second timepoint (T2 samples) with a median time of 10-months (interquartile range: 5-months) (see **Table 3**).

**Table 2:** Demographics of study population.

	Natalizumab Patients (n = 22)	AHSCT Patients (n = 8)
Age (years)		
Median ± SD	36.5 ± 10.8	37.5 ± 6.7
Sex, n (%)		
Male	2 (9.1)	2 (25.0)
Female	20 (90.9)	6 (75.0)
Ethnicity (%)		
Anglo-Celtic	15 (68.2)	7 (87.5)
European	4 (18.2)	0 (0)
East/South-East Asian	1 (4.5)	0 (0)
Aboriginal	0 (0)	1 (12.5)
Middle Eastern	2 (9.1)	0 (0)
Type of MS (%)		
Relapsing Remitting	21 (95.5)	8 (100)
Secondary Progressive	1 (4.5)	0 (0)
EDSS		
Median ± SD	2.0 ± 0.9	4.8 ± 1.7
Smoking, n (%)		
Smoker	0 (0)	0 (0)
Non-smoker	22 (100)	8 (100)
Alcohol, n (%)		
>3 standard drinks per week	5 (22.7)	0 (0)
<3 standard drinks per week	17 (77.3)	8 (100)
Antibiotics used in last 12 months, n (%)		
Yes	6 (27.3)	7 (87.5)*
No	16 (72.7)	1 (12.5)

*Refers to AHSCT patients taking antibiotics 12 months prior to treatment and baseline (T1) sample collection. No AHSCT patients received natalizumab in the 6 months prior to receiving AHSCT. Additionally, all patients in the AHSCT cohort had antimicrobial prophylaxis for 6-months post-transplant; with trimethoprim/sulfamethoxazole, valaciclovir and fluconazole.

**Table 3:** Longitudinal analysis, T1 and T2 sample information.

Patient Number	Timepoint 1 (Stool and Oral) Sample	Timepoint 2 (Stool and Oral) Sample
Patient 1	Baseline (pre-transplant)	7 months
Patient 2	Baseline (pre-transplant)	7 months
Patient 3	Baseline (pre-transplant)	12 months
Patient 4	Baseline (pre-transplant)	12 months
Patient 5	Baseline (pre-transplant)	10 months
Patient 6	Baseline (pre-transplant)	10 months

All patients were part of the AHSCT cohort. Stool and oral samples were collected at the same time. Median timepoint for 2nd sample = 10 months, interquartile range = 5 months.

### 3.2 Comparison of baseline oral and stool microbiota

**Figure 1:**
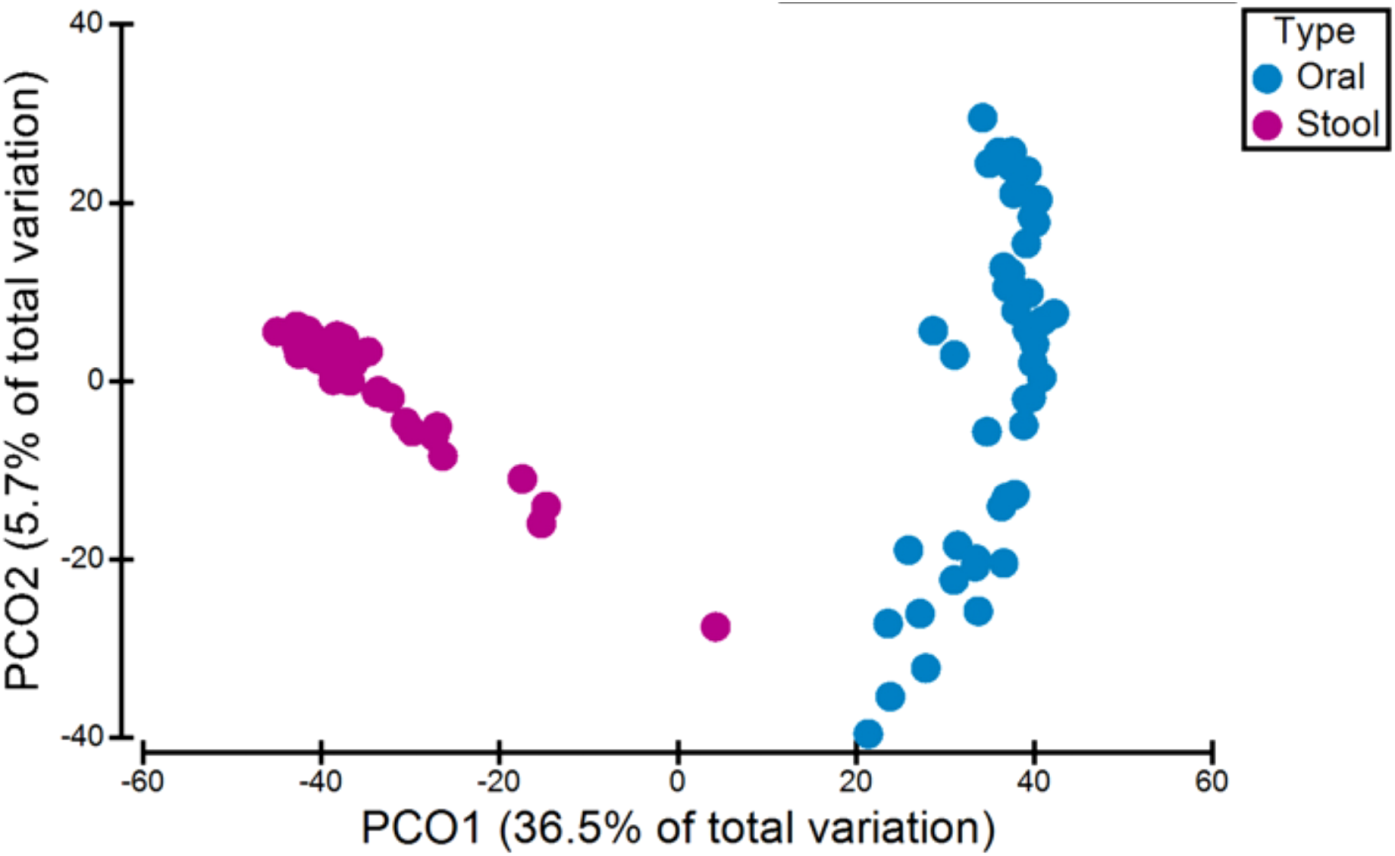
Variation in microbiota (beta-diversity) between all oral and stool samples. There was a significant difference between oral and stool microbiota (p=0.0001, PERMANOVA).

To analyse the overall interindividual difference between stool and oral samples (beta diversity), PCoA and a non-parametric multivariate statistical permutation test (PERMANOVA) was performed. There was a highly significant difference between oral and stool microbiota (p=0.0001, PERMANOVA) (see **Figure 1**).

### 3.3 AHSCT vs NTZ cohorts

**Figure 2:**
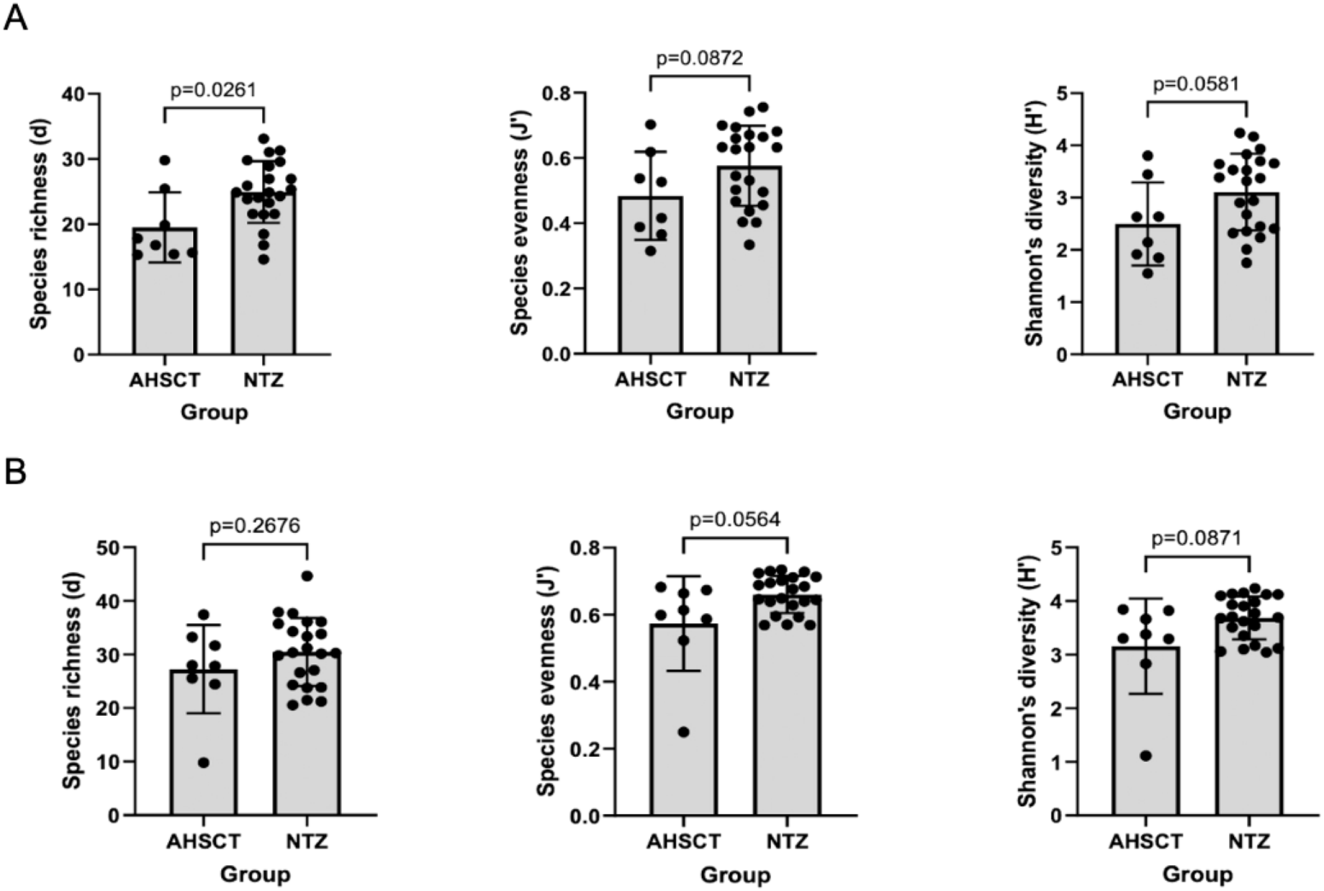
**(A)** Comparison of oral alpha diversity between AHSCT and NTZ cohort; **(B)** Comparison of stool alpha diversity between AHSCT and NTZ cohorts. Normality of datasets was determined using Shapiro-Wilk tests. Unpaired t-tests (parametric) or Mann-Whitney U tests (non-parametric) were then used to compare alpha diversity statistics, including Margalef’s richness, Pielou’s evenness and Shannon’s diversity. For the oral samples, there was a trend towards lower alpha diversity in the AHSCT cohorts. However, only species richness was statistically significant (p=0.0261).

**Figure 3:**
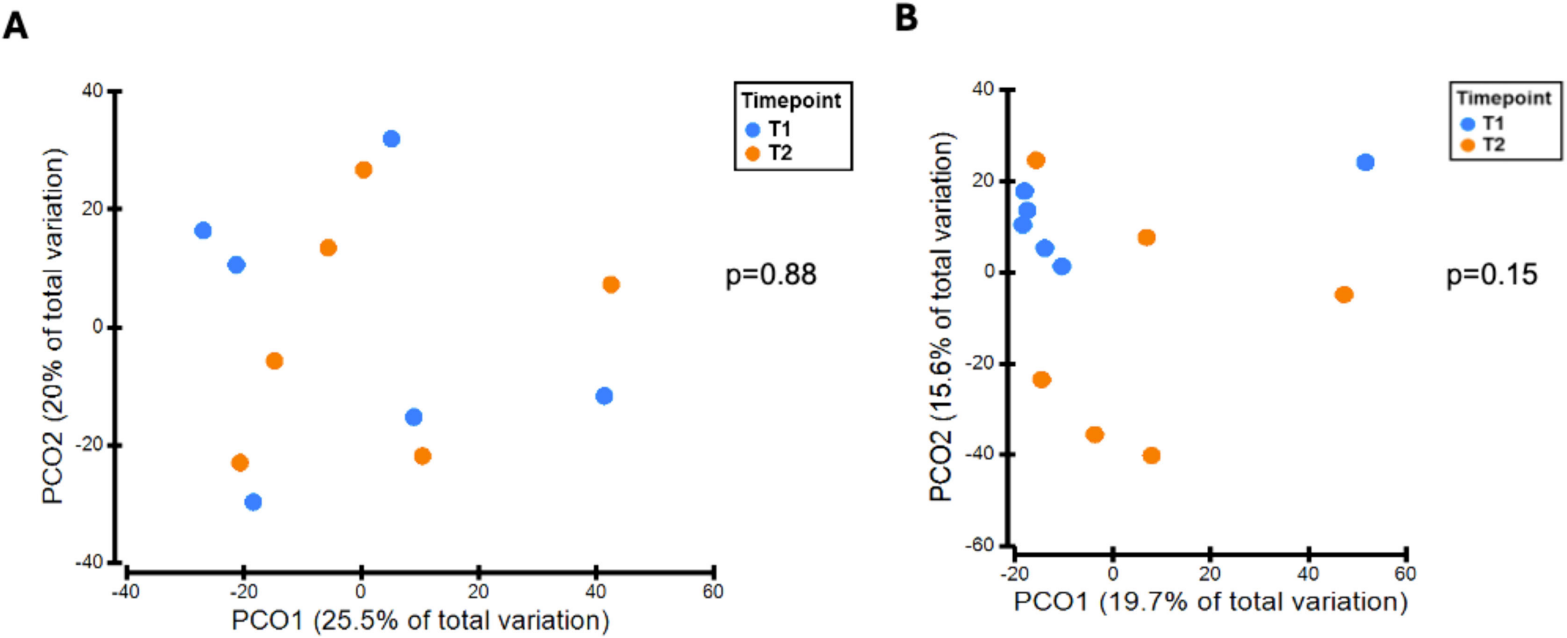
**(A)** Oral microbiota variation between AHSCT and NTZ cohorts, **(B)** Stool microbiota variation between AHSCT and NTZ cohorts. Beta diversity statistics were generated using PCoA and PERMANOVA. PCoA was performed on Bray Curtis resemblance matrix of square root transformed relative abundances. There were significant differences in beta-diversity for oral (p=0.043, PERMANOVA) and stool (p=0.0086, PERMANOVA) samples between AHSCT and NTZ cohorts.

The AHSCT cohort had significantly lower species richness of oral microbiota compared to the NTZ cohort (p=0.026) (**Figure 2A**). While there was no significant difference in evenness (p=0.0872) and diversity (p=0.0581) between the two cohorts, there was a trend to lower evenness and diversity in the AHSCT group. While generally lower in the AHSCT cohort, there was no significant difference in the alpha diversity of the stool microbiota richness (p=0.2676), evenness (p=0.0564) and diversity (p=0.0871) between the AHSCT and NTZ cohorts (**Figure 2B**). There was a significant difference in the oral beta diversity (p=0.043, PERMANOVA) (**Figure 3A**) and stool beta diversity (p=0.0086, PERMANOVA) (**Figure 3B**) between the AHSCT and NTZ groups.

### 3.4 Longitudinal AHSCT analysis

**Figure 4:**
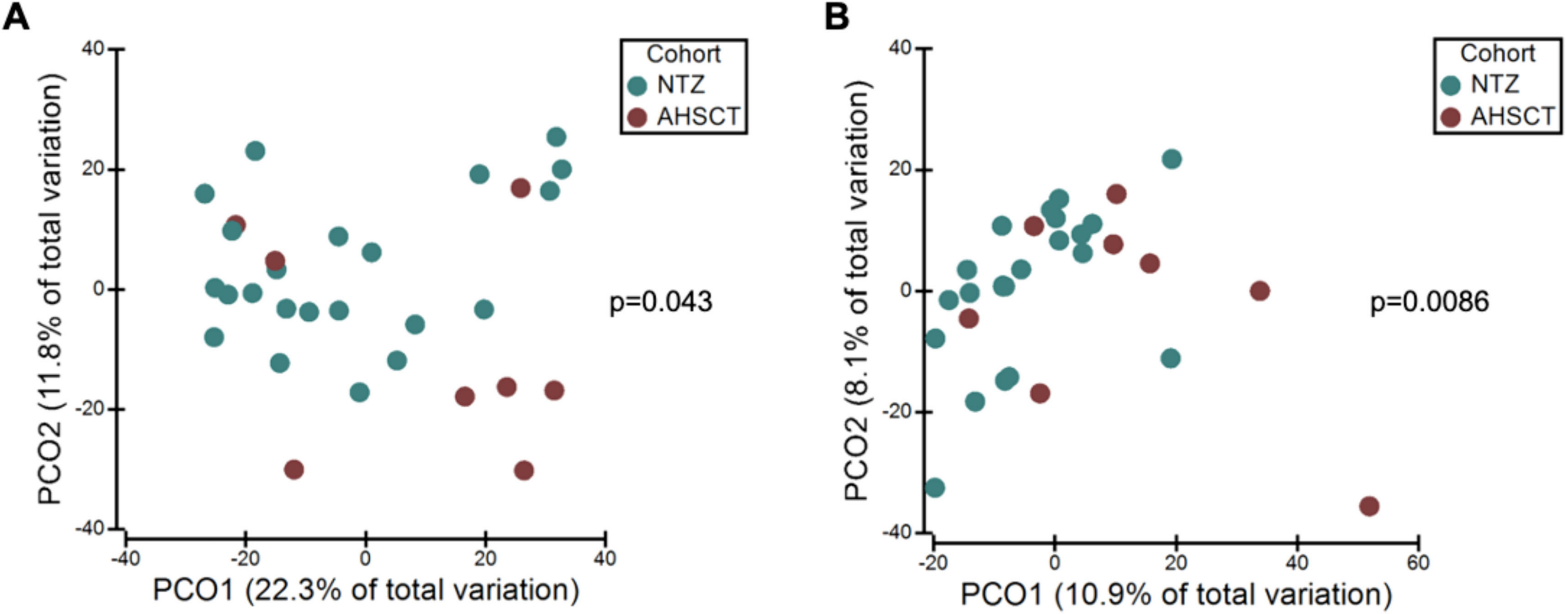
**(A)** Comparison of oral alpha diversity between T1 and T2 samples, **(B)** Comparison of stool alpha diversity between T1 and T2 samples. The differences in alpha diversity, including Margalef’s richness, Pielou’s evenness and Shannon’s diversity, between T1 and T2 samples was determined using unpaired t-tests and Mann Whitney U tests. Analysis of both oral and stool microbiota revealed no differences in alpha diversity between T1 and T2 samples.

**Figure 5:**
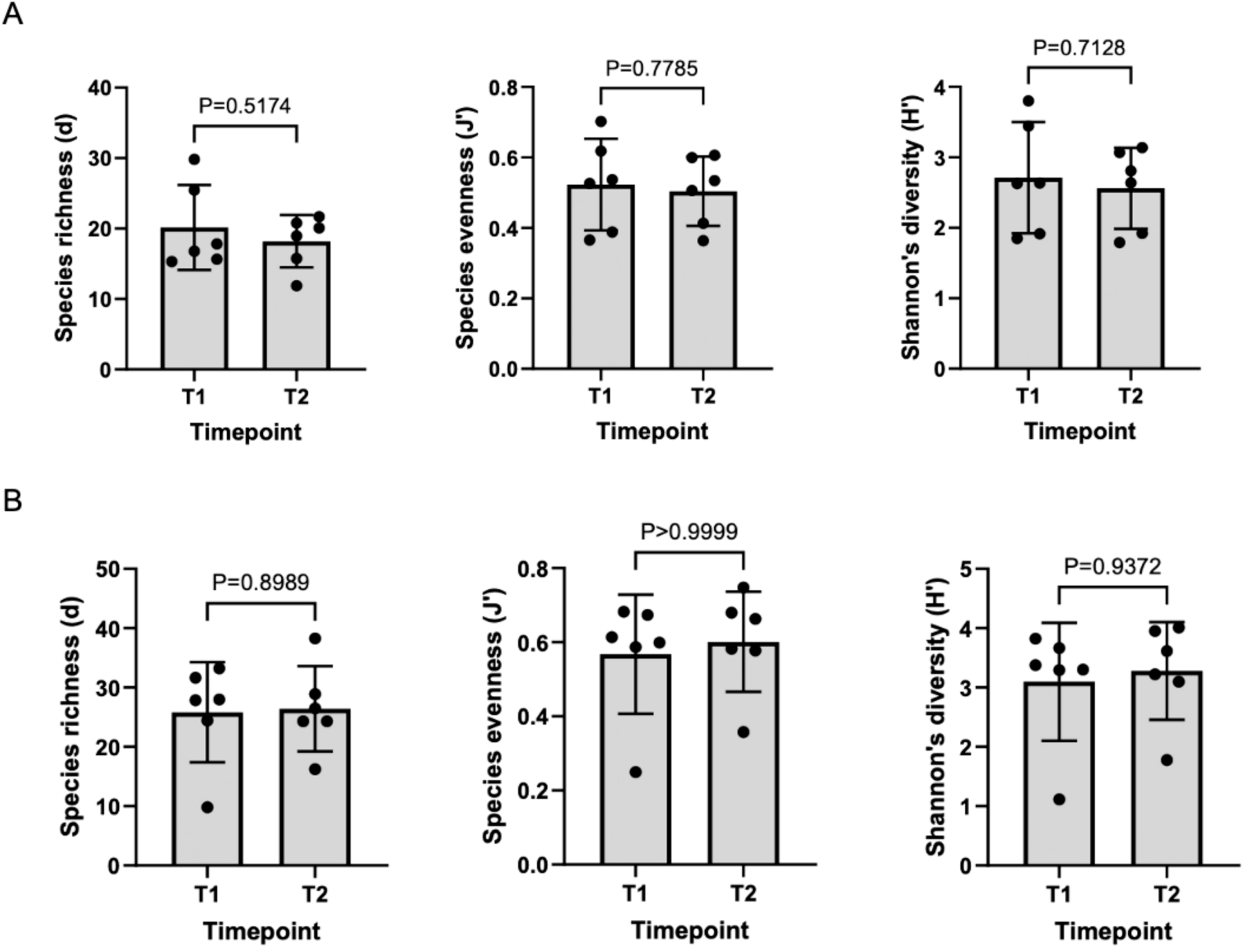
**(A)** Variation in oral microbiota between T1 and T2 samples, **(B)** Variation in stool microbiota between T1 and T2 samples. There was no significant difference in oral (p=0.88, PERMANOVA) or stool (p=0.15, PERMANOVA) beta-diversity between T1 and T2 samples.

There was no significant difference in alpha diversity between T1 and T2 oral microbiota samples (**Figure 4A**). T1 and T2 stool microbiota samples also revealed no significant differences in alpha diversity (**Figure 4B**). Comparison of oral samples at T1 and T2 showed no differences in beta diversity (p=0.88, PERMANOVA) (**Figure 5A**). Similarly, comparison of stool samples at T1 and T2 showed no differences in beta diversity (p=0.15, PERMANOVA) (**Figure 5B**).

### 3.5 Taxa analysis of NTZ and AHSCT cohorts at baseline

A per taxa analysis was performed to identify operational taxonomy units (OTUs) which contributed to differences in beta diversity between AHSCT and NTZ cohorts. The taxa were identified at the genus level, their lowest possible level of classification (**Table 4**). For stool samples, the AHSCT group had greater relative abundances of an unclassified genus of Lachnospiraceae, Enterocloster, Bifidobacterium and Parabacteroides and lower abundances of Roseburia and Hominimerdicola. For oral microbiota, the AHSCT group has greater relative abundances of Porphyromonas and decreased abundances of Veillonella.

**Table 4:** Summary of oral and stool OTUs.

*Oral Microbiota*			
OTUs	q-value corrected for FDR	Fold Change AHSCT/NTZ	Bacterial species
Porphyromonas_OTU69	0.015	1.16	Gram-negative anaerobe associated with numerous systemic inflammatory and neurodegenerative disease (26).
Veillonella_OTU2	0.100	0.27	Gram-negative obligate anaerobe (27).
*Stool Microbiota*			
Lachnospiraceae_unclassified_OTU406	0.032	3.39	Gram-positive anaerobe associated with SCFA production (28).
Enterocloster_OTU121	0.056	5.37	Gram-positive obligate anaerobe associated with SCFA production (28).
Roseburia_OTU68	0.089	0.16	Gram-positive obligate anaerobe associated with SCFA production (28).
Bifidobacterium_OTU405	0.089	2.10	Gram-positive, facultative anaerobe associated with SCFA production (28).
Parabacteroides_OTU15	0.091	1.75	Gram-negative anaerobe involved in SCFA production (29).
Hominimerdicola_OTU25	0.091	0.20	Gram-positive obligate anaerobe.

The Q-value corrected for the false discovery rate (FDR) adjusts the P-value to control for the proportion of false positives among the significant results. The oral and stool OTUs are ordered from most to least statistically significant based of the q-values. ‘Fold change AHSCT/NTZ’ indicates the relative abundance of a specific OTU in the AHSCT group compared to the NTZ group. A number >1 indicates the OTU is more abundant in the AHSCT group and vice versa.

OTU, operational taxonomy units, SCFA, short-chain fatty acid.

## 4 Discussion

### 4.1 Comparison of AHSCT and NTZ cohorts

Despite emerging evidence linking oral bacteria to various systemic diseases (30), the role of oral microbiota in MS remains underexplored. While previous research predominantly compares individuals with MS to healthy controls (HC) (16–21), this study offers a novel approach by examining microbiome differences across MS patients with differing treatment exposures and disease activity levels. Specifically, we focused on patients undergoing AHSCT, who typically have active, treatment-refractory MS, and compared them to patients receiving NTZ, with controlled, non-active MS.

A key finding in this study was the lower species richness of oral microbiota in the ASHCT group compared to the NTZ group. The correlation of reduced alpha diversity in the more active AHSCT cohort raises the possibility that increased oral diversity may have a protective effect in MS. This supports the observations of a longitudinal study comparing the microbiome of pwMS undergoing ocrelizumab therapy. The study reported decreased oral alpha diversity in MS patients compared to HC, revealing that diversity patterns were most pronounced in patients with higher disease activity and disability (31). It is unclear whether these oral microbial changes are an effect of MS therapy or indirectly modulate MS disease activity and this requires further investigation.

Our findings of increased oral Porphyromonas in the AHSCT group is also notable, given previous studies have reported an increased abundance of oral Porphyromonas in pwMS compared to HC (32, 33). Additionally, Porphyromonas gingivalis has been associated with several systemic diseases, including Alzheimer’s disease, various cancers, atherosclerosis, periodontitis, and systemic inflammation (34–36). Its pathogenic potential can be attributed to its lipopolysaccharides, particularly the lipid A structure, which can trigger proinflammatory responses by activating the NF-κB signalling pathways and promoting the secretion of inflammatory cytokines (37). In EAE, a murine model of demyelination, when immune-competent glial cells of the CNS are exposed to antigens of Porphyromonas gingivalis, immune responses and disability scores are exacerbated. However, the significance of this finding needs to be confirmed in human studies (38, 39).

We observed a significant depletion of oral Veillonella in ‘active’ MS (pre-AHSCT cohort) compared to treated MS (NTZ group). A recent study comparing pwMS to HC found increased abundance of oral Veillonella in MS populations. Several factors may explain these divergent findings. Veillonella is part of the core oral microbiome in healthy individuals and its abundance can be influenced by confounders such as periodontal disease, oral hygiene, and dietary habits (40, 41). Notably, a greater proportion of AHSCT patients had received antibiotics in the 12 months prior to sample collection, which may have contributed to the observed depletion, as systemic antibiotic use is known to disrupt oral microbial communities even when not directly targeting the oral cavity (42). It is also important to consider that our comparison involves two MS subgroups with differing disease activity and treatment exposure, rather than a comparison with healthy controls. As such, the observed differences in Veillonella abundance may reflect treatment effects, immunological state, or other systemic influences. Thus, further studies with larger MS cohorts with controlled oral confounders are required to are required to clarify Veillonella’s role and clinical relevance in the context of MS.

Our study revealed no differences in stool alpha diversity between AHSCT and NTZ groups. Similar results were observed in the iMSMS study (21) which found no differences in alpha diversity between treated and non-treated MS populations. Beta diversity between the AHSCT and NTZ cohorts, however, was significantly different, highlighting the possibility that varying MS disease states may be associated with stool microbial composition.

Specific taxa findings revealed increased relative abundances of an unclassified genus of Lachnospiraceae, Enterocloster, Bifidobacterium and Parabacteroides in the AHSCT group compared to the NTZ group. While the lack of HCs in this study makes it difficult to confirm whether these bacteria are significantly altered in MS populations compared to healthy individuals, evidence from numerous studies show they are likely to be implicated in MS (18, 20, 21, 43–45). Consistent with previous studies, we found lower abundances of Roseburia and Hominimerdicola in the AHSCT group. Roseburia has been noted to decrease in MS patients compared to healthy controls (45, 46), suggesting a protective role in MS. It is hypothesised that Roseburia may ameliorate MS through its production of the butyrate, an SCFA with anti-inflammatory properties (47). It should be acknowledged that prior work has conversely suggested that Lachnospiraceae, Bifidobacteirum and Parabacteroides have decreased abundance in untreated MS populations compared to HC (43–45). Specifically, Parabacteroides distasonis has been shown to induce regulatory T cells that produce anti-inflammatory IL-10 (48). Acetate, a SCFA highly produced by Bifidobacteirum, regulates intestinal inflammation by stimulating the GPR43 receptor which in turn, inhibits the secretion of pro-inflammatory IL-18 (49). These conflicting results highlight the complex relationship between specific bacterial taxa, MS and the immune system which can be modulated by other factors, such as disease stage and treatment status.

Interestingly, a consistent pattern emerging from both our study and previous research is the association of MS with SCFA-producing bacteria (46). Five out of the six taxa identified in our study responsible for the stool beta-diversity differences are involved in the production of SCFAs. SCFAs, particularly butyrate and acetate, can promote anti-inflammatory responses by supporting regulatory T cells but also influence pro-inflammatory Th1 and Th17 cell differentiation in certain contexts (50, 51). Increased acetate levels in MS patients correlated with higher disability levels measured by their EDSS (52). While our findings highlight a strong association between stool microbiota and their metabolites with MS, further investigation into bacterial metabolites like SCFAs in MS populations is required to elucidate the exact mechanisms involved.

A potential confounding factor is the effect of NTZ on both oral and gut microbiota. Currently, there is limited evidence on the impact of NTZ on oral microbial composition. However, research has demonstrated that NTZ treatment can reduce specific stool bacterial populations, including some that produce short-chain fatty acids (SCFAs), such as Bacteroides and Bifidobacterium species, which are key to gastrointestinal health (21, 53). While it is believed NTZ’s mechanism of action against MS involves the inhibition of T-cell trafficking to the CNS, it also impacts T-cell circulation within the GIT (54). This immunomodulatory action on the gut could influence microbial composition, possibly representing an alternative pathway by which this therapy acts against MS. NTZ-treated patients were chosen as the comparator group due to their clinical stability, consistent treatment duration, and lack of recent immunosuppressive induction, which make them a valuable reference for understanding microbiome profiles in MS under controlled treatment conditions. However, the differences in microbial composition between the AHSCT and NTZ cohorts may be due to treatment-specific effects, in addition to or instead of disease activity.

### 4.2 Longitudinal analysis

Analysis of T1 (pre-transplant) and T2 (post-transplant) samples for both oral and stool demonstrated no significant differences in alpha- or beta-diversity, revealing that the diversity of stool and oral microbial populations remained relatively stable post-AHSCT, despite the peri-transplant use of chemotherapy, steroids and antimicrobials. Our findings contrast numerous studies where a marked reduction in stool alpha diversity post-allogenic HSCT was observed (55, 56). In Khan et al., the largest and first multicentre study of stool microbiota composition in autologous HSCT, alpha diversity of post-transplant stool samples was also significantly decreased (57). A plausible explanation for the discrepancy in our results lies in the different post-transplant timeframes assessed across studies. While the aforementioned studies collected post-transplant stool samples within the at 0–100 days post-transplant, our study examined samples collected between 7–12 months post-transplant. This extended period may have allowed the stool microbial population to recover and return to pre-transplant diversity levels, and this return to ‘baseline’ is a fascinating observation in itself. This is supported by longitudinal data from Khan et al., where microbial diversity in stool samples reached its lowest point at 14–17 days post-transplant but subsequently increased (57).

The diversity of gut microbiome before and after transplant has also been linked to improved clinical outcomes. Notably, research has indicated that higher gut microbiota diversity pre-transplant correlates with improved overall survival and a lower incidence of acute graft-versus-host disease (GVHD) in allogeneic HSCT patients (58–60). Additionally, lower diversity during the peri-engraftment phase has been associated with worse outcomes in autologous HSCT patients (57). While these correlations have primarily been drawn from malignant-related HSCT cases, they suggest that the stability of the gut microbiome post-AHSCT in our cohort of MS patients may be linked to positive clinical outcomes, especially since all patients our AHSCT cohort have met the “no evidence of disease activity” (NEDA) criteria and have not progressed in terms of MS disability. However, a larger sample size and correlation to long-term follow up data is required to validate such relationships.

### 4.3 Limitations and future direction

This study was limited by the relatively small sample size of 30 patients. In particular, recruitment for the AHSCT cohort was restricted by the number of MS patients who met the inclusion criteria for the AiMS study at St Vincent’s Hospital, and ongoing challenges through 2022 with COVID-19. On average, only 5–10 patients undergo AHSCT for MS each year, which has limited the pace of recruitment. As this pilot study is ongoing, we anticipate that a more comprehensive analysis of longitudinal changes in the oral and gut microbiome will be possible as additional data are collected across multiple timepoints. These future analyses may enable correlation of microbial profiles with clinical outcomes, including MS severity and markers of immune reconstitution following AHSCT.

Although the NTZ and AHSCT cohorts were broadly matched for age, sex, and ethnicity, minimising the likelihood of major demographic confounding, statistical analyses to explore associations between individual demographic variables and microbiota composition were not conducted. This was due to the limited sample size and lack of statistical power for reliable subgroup analysis but can also be addressed as additional data is collected.

Due to small numbers, confounding factors in each cohort such as gender, diet or recent antibiotics couldn’t be accounted for and may influence GIT microbial composition. Oral health parameters such as periodontal status and oral hygiene practices were not assessed. Given the known influence of these factors on oral microbial composition, their omission may have impacted our findings (61–63). In future studies, adopting a household paired system whereby the patient with MS and controls live in the same household could address this issue as both individuals would have similar diets and lifestyle factors. Incorporating standardised oral health assessments may also better control for oral microbiome variability.

The variability in timing of post-AHSCT sample collection (between 7–12 months) is also acknowledged as a limitation. This arose due to the logistics of home sampling and postal return. While all samples were collected after the acute transplant phase, this range may have influenced microbiota composition. As more data are collected, we intend to stratify post-AHSCT samples into narrower timepoints (e.g. 6–8 months vs 9–12 months) to examine trends with greater granularity.

Additionally, Natalizumab’s effects on immune cell trafficking in the gastrointestinal tract may independently influence microbiota composition. This introduces a potential confounder when interpreting differences between cohorts and highlights the need for future studies incorporating additional control groups.

This study did not aim to compare MS cohorts with a healthy control population, as this has been extensively previously explored. However, the lack of an internal control is another limitation in our study. Consequently, it was questionable whether the differences between our two cohorts were due to the differing treatments or the varying severity of disease. However, this limitation was partially overcome by supporting evidence from studies which found that the specific bacterial taxa identified in our study were previously implicated in MS.

## 5 Conclusion

This pilot study identified specific microbiome changes, particularly in the oral alpha diversity and abundance of specific bacteria which may play roles in MS progression or pathogenesis. Furthermore, longitudinal analysis combined with clinical follow-up data alludes to a potential relationship between stool microbial diversity and positive AHSCT outcomes. Ongoing recruitment and further analysis will determine the validity of these findings and may provide a novel therapeutic target in MS treatment.

## Data Availability

The raw data supporting the conclusions of this article will be made available by the authors, without undue reservation.

## Appendix 1

### Washout periods off DMT prior to sample collection

- S1P receptor modulators (i.e. Fingolimod): 4 weeks from last dose
- Cladribine: 6 months from last dose
- Natalizumab: 6 weeks from last dose
- CD20 monoclonal antibodies (Ocrelizumab/Ofatumumab): 3 months from last dose
- Alemtuzumab: 6 months from last dose

## References

[1] Walton C King R Rechtman L Kaye W Leray E Marrie RA. Rising prevalence of multiple sclerosis worldwide: Insights from the Atlas of MS, third edition. Mult Scler. (2020) 26:1816–21. doi: 10.1177/1352458520970841 PMC7720355 33174475

[2] Habbestad A Willumsen JS Aarseth JH Grytten N Midgard R Wergeland S. Increasing age of multiple sclerosis onset from 1920 to 2022: a population-based study. J Neurol. (2024) 271:1610–7. doi: 10.1007/s00415-023-12047-9 PMC10973050 38097800

[3] Ford H. Clinical presentation and diagnosis of multiple sclerosis. Clin Med (Lond). (2020) 20:380–3. doi: 10.7861/clinmed.2020-0292 PMC7385797 32675142

[4] Moutsianas L Jostins L Beecham AH Dilthey AT Xifara DK Ban M. Class II HLA interactions modulate genetic risk for multiple sclerosis. Nat Genet. (2015) 47:1107–13. doi: 10.1038/ng.3395 PMC4874245 26343388

[5] Hollenbach JA Oksenberg JR. The immunogenetics of multiple sclerosis: A comprehensive review. J Autoimmun. (2015) 64:13–25. doi: 10.1016/j.jaut.2015.06.010 26142251 PMC4687745

[6] Berer K Krishnamoorthy G. Microbial view of central nervous system autoimmunity. FEBS Lett. (2014) 588:4207–13. doi: 10.1016/j.febslet.2014.04.007 24746689

[7] Massey JC Sutton IJ Ma DDF Moore JJ. Regenerating immunotolerance in multiple sclerosis with autologous hematopoietic stem cell transplant. Front Immunol. (2018) 9:410. doi: 10.3389/fimmu.2018.00410 29593711 PMC5857574

[8] Voet S Prinz M van Loo G. Microglia in central nervous system inflammation and multiple sclerosis pathology. Trends Mol Med. (2019) 25:112–23. doi: 10.1016/j.molmed.2018.11.005 30578090

[9] Biswas S Bryant RV Travis S. Interfering with leukocyte trafficking in Crohn’s disease. Best Pract Res Clin Gastroenterol. (2019) 38-39:101617. doi: 10.1016/j.bpg.2019.05.004 31327399

[10] Nguyen Ky M Duran A Hasantari I Bru A Deloire M Brochet B. Natalizumab treatment induces proinflammatory CD4 T cells preferentially in the integrin β7+ Compartment. Neurol Neuroimmunol Neuroinflamm. (2023) 10. doi: 10.1212/NXI.0000000000200166 PMC10519437 37739811

[11] Cohen JA Baldassari LE Atkins HL Bowen JD Bredeson C Carpenter PA. Autologous hematopoietic cell transplantation for treatment-refractory relapsing multiple sclerosis: position statement from the american society for blood and marrow transplantation. Biol Blood Marrow Transpl. (2019) 25:845–54. doi: 10.1016/j.bbmt.2019.02.014 30794930

[12] Chen Y Xu J Chen Y. Regulation of neurotransmitters by the gut microbiota and effects on cognition in neurological disorders. Nutrients. (2021) 13. doi: 10.3390/nu13062099 PMC8234057 34205336

[13] Fülling C Dinan TG Cryan JF. Gut microbe to brain signaling: what happens in vagus…. Neuron. (2019) 101:998–1002. doi: 10.1016/j.neuron.2019.02.008 30897366

[14] Ivanov II Atarashi K Manel N Brodie EL Shima T Karaoz U. Induction of intestinal Th17 cells by segmented filamentous bacteria. Cell. (2009) 139:485–98. doi: 10.1016/j.cell.2009.09.033 PMC2796826 19836068

[15] Correale J Hohlfeld R Baranzini SE. The role of the gut microbiota in multiple sclerosis. Nat Rev Neurol. (2022) 18:544–58. doi: 10.1038/s41582-022-00697-8 35931825

[16] Cantarel BL Waubant E Chehoud C Kuczynski J DeSantis TZ Warrington J. Gut microbiota in multiple sclerosis: possible influence of immunomodulators. J Investig Med. (2015) 63:729–34. doi: 10.1097/JIM.0000000000000192 PMC4439263 25775034

[17] Miyake S Kim S Suda W Oshima K Nakamura M Matsuoka T. Dysbiosis in the gut microbiota of patients with multiple sclerosis, with a striking depletion of species belonging to clostridia XIVa and IV clusters. PloS One. (2015) 10:e0137429. doi: 10.1371/journal.pone.0137429 26367776 PMC4569432

[18] Chen J Chia N Kalari KR Yao JZ Novotna M Paz Soldan MM. Multiple sclerosis patients have a distinct gut microbiota compared to healthy controls. Sci Rep. (2016) 6:28484. doi: 10.1038/srep28484 27346372 PMC4921909

[19] Jangi S Gandhi R Cox LM Li N von Glehn F Yan R. Alterations of the human gut microbiome in multiple sclerosis. Nat Commun. (2016) 7:12015. doi: 10.1038/ncomms12015 27352007 PMC4931233

[20] Berer K Gerdes LA Cekanaviciute E Jia X Xiao L Xia Z. Gut microbiota from multiple sclerosis patients enables spontaneous autoimmune encephalomyelitis in mice. Proc Natl Acad Sci U S A. (2017) 114:10719–24. doi: 10.1073/pnas.1711233114 PMC5635914 28893994

[21] Zhou X Baumann R Gao X Mendoza M Singh S Katz Sand I. Gut microbiome of multiple sclerosis patients and paired household healthy controls reveal associations with disease risk and course. Cell. (2022) 185:3467–86.e16. doi: 10.1016/j.cell.2022.08.021 36113426 PMC10143502

[22] Thompson AJ Banwell BL Barkhof F Carroll WM Coetzee T Comi G. Diagnosis of multiple sclerosis: 2017 revisions of the McDonald criteria. Lancet Neurol. (2018) 17:162–73. doi: 10.1016/S1474-4422(17)30470-2 29275977

[23] Doki N Suyama M Sasajima S Ota J Igarashi A Mimura I. Clinical impact of pre-transplant gut microbial diversity on outcomes of allogeneic hematopoietic stem cell transplantation. Ann Hematol. (2017) 96:1517–23. doi: 10.1007/s00277-017-3069-8 28733895

[24] Schloss PD Westcott SL Ryabin T Hall JR Hartmann M Hollister EB. Introducing mothur: open-source, platform-independent, community-supported software for describing and comparing microbial communities. Appl Environ Microbiol. (2009) 75:7537–41. doi: 10.1128/AEM.01541-09 PMC2786419 19801464

[25] Yang L Chen J. A comprehensive evaluation of microbial differential abundance analysis methods: current status and potential solutions. Microbiome. (2022) 10:130. doi: 10.1186/s40168-022-01320-0 35986393 PMC9392415

[26] Olsen I. Porphyromonas gingivalis-induced neuroinflammation in Alzheimer’s disease. Front Neurosci. (2021) 15:691016. doi: 10.3389/fnins.2021.691016 34720846 PMC8551391

[27] Retnakumar RJ Nath AN Nair GB Chattopadhyay S. Chapter Three - Gastrointestinal microbiome in the context of Helicobacter pylori infection in stomach and gastroduodenal diseases. Prog Mol Biol Transl Sci. (2022) 192:53–95. doi: 10.1016/bs.pmbts.2022.07.001 36280325

[28] Fusco W Lorenzo MB Cintoni M Porcari S Rinninella E Kaitsas F. Short-chain fatty-acid-producing bacteria: key components of the human gut microbiota. Nutrients. (2023) 15. doi: 10.3390/nu15092211 PMC10180739 37432351

[29] Cui Y Zhang L Wang X Yi Y Shan Y Liu B. Roles of intestinal Parabacteroides in human health and diseases. FEMS Microbiol Lett. (2022) 369. doi: 10.1093/femsle/fnac072 35945336

[30] Peng X Cheng L You Y Tang C Ren B Li Y. Oral microbiota in human systematic diseases. Int J Oral Sci. (2022) 14:14. doi: 10.1038/s41368-022-00163-7 35236828 PMC8891310

[31] Troci A Zimmermann O Esser D Krampitz P May S Franke A. B-cell-depletion reverses dysbiosis of the microbiome in multiple sclerosis patients. Sci Rep. (2022) 12:3728. doi: 10.1038/s41598-022-07336-8 35260584 PMC8904534

[32] Boussamet L Montassier E Mathé C Garcia A Morille J Shah S. Investigating the metabolite signature of an altered oral microbiota as a discriminant factor for multiple sclerosis: a pilot study. Sci Rep. (2024) 14:7786. doi: 10.1038/s41598-024-57949-4 38565581 PMC10987558

[33] Zangeneh Z Abdi-Ali A Khamooshian K Alvandi A Abiri R. Bacterial variation in the oral microbiota in multiple sclerosis patients. PloS One. (2021) 16:e0260384. doi: 10.1371/journal.pone.0260384 34847159 PMC8631616

[34] Rafiei M Kiani F Sayehmiri F Sayehmiri K Sheikhi A Zamanian Azodi M. Study of Porphyromonas gingivalis in periodontal diseases: A systematic review and meta-analysis. Med J Islam Repub Iran. (2017) 31:62. doi: 10.14196/mjiri.31.62 29445691 PMC5804457

[35] Gnanasekaran J Binder Gallimidi A Saba E Pandi K Eli Berchoer L Hermano E. Intracellular porphyromonas gingivalis promotes the tumorigenic behavior of pancreatic carcinoma cells. Cancers (Basel). (2020) 12. doi: 10.3390/cancers12082331 PMC7465784 32824786

[36] Dominy SS Lynch C Ermini F Benedyk M Marczyk A Konradi A. Porphyromonas gingivalis in Alzheimer’s disease brains: Evidence for disease causation and treatment with small-molecule inhibitors. Sci Adv. (2019) 5:eaau3333. doi: 10.1126/sciadv.aau3333 30746447 PMC6357742

[37] Nativel B Couret D Giraud P Meilhac O d’Hellencourt CL Viranaïcken W. Porphyromonas gingivalis lipopolysaccharides act exclusively through TLR4 with a resilience between mouse and human. Sci Rep. (2017) 7:15789. doi: 10.1038/s41598-017-16190-y 29150625 PMC5693985

[38] Shapira L Ayalon S Brenner T. Effects of porphyromonas gingivalis on the central nervous system: activation of glial cells and exacerbation of experimental autoimmune encephalomyelitis. J Periodontol. (2002) 73:511–6. doi: 10.1902/jop.2002.73.5.511 12027253

[39] Memedovski Z Czerwonka E Han J Mayer J Luce M Klemm LC. Classical and alternative activation of rat microglia treated with ultrapure porphyromonas gingivalis lipopolysaccharide *in vitro* . Toxins (Basel). (2020) 12. doi: 10.3390/toxins12050333 PMC7290770 32438602

[40] Perera M Al-hebshi NN Speicher DJ Perera I Johnson NW. Emerging role of bacteria in oral carcinogenesis: a review with special reference to perio-pathogenic bacteria. J Oral Microbiol. (2016) 8:32762. doi: 10.3402/jom.v8.32762 27677454 PMC5039235

[41] Li X Liu Y Yang X Li C Song Z. The oral microbiota: community composition, influencing factors, pathogenesis, and interventions. Front Microbiol. (2022) 13:895537. doi: 10.3389/fmicb.2022.895537 35572634 PMC9100676

[42] Cheng X He F Si M Sun P Chen Q. Effects of antibiotic use on saliva antibody content and oral microbiota in sprague dawley rats. Front Cell Infect Microbiol. (2022) 12. doi: 10.3389/fcimb.2022.721691 PMC8843035 35174102

[43] Cekanaviciute E Yoo BB Runia TF Debelius JW Singh S Nelson CA. Gut bacteria from multiple sclerosis patients modulate human T cells and exacerbate symptoms in mouse models. Proc Natl Acad Sci. (2017) 114:10713–8. doi: 10.1073/pnas.1711235114 PMC5635915 28893978

[44] Cantoni C Lin Q Dorsett Y Ghezzi L Liu Z Pan Y. Alterations of host-gut microbiome interactions in multiple sclerosis. EBioMedicine. (2022) 76:103798. doi: 10.1016/j.ebiom.2021.103798 35094961 PMC8814376

[45] Ling Z Cheng Y Yan X Shao L Liu X Zhou D. Alterations of the fecal microbiota in chinese patients with multiple sclerosis. Front Immunol. (2020) 11:590783. doi: 10.3389/fimmu.2020.590783 33391265 PMC7772405

[46] Saresella M Marventano I Barone M La Rosa F Piancone F Mendozzi L. Alterations in circulating fatty acid are associated with gut microbiota dysbiosis and inflammation in multiple sclerosis. Front Immunol. (2020) 11:1390. doi: 10.3389/fimmu.2020.01390 32733460 PMC7358580

[47] Tamanai-Shacoori Z Smida I Bousarghin L Loreal O Meuric V Fong SB. Roseburia spp.: A marker of health? Future Microbiol. (2017) 12:157–70. doi: 10.2217/fmb-2016-0130 28139139

[48] Ezeji JC Sarikonda DK Hopperton A Erkkila HL Cohen DE Martinez SP. Parabacteroides distasonis: intriguing aerotolerant gut anaerobe with emerging antimicrobial resistance and pathogenic and probiotic roles in human health. Gut Microbes. (2021) 13:1922241. doi: 10.1080/19490976.2021.1922241 34196581 PMC8253142

[49] Maslowski KM Vieira AT Ng A Kranich J Sierro F Yu D. Regulation of inflammatory responses by gut microbiota and chemoattractant receptor GPR43. Nature. (2009) 461:1282–6. doi: 10.1038/nature08530 PMC3256734 19865172

[50] Li M van Esch BCAM Wagenaar GTM Garssen J Folkerts G Henricks PAJ. Pro- and anti-inflammatory effects of short chain fatty acids on immune and endothelial cells. Eur J Pharmacol. (2018) 831:52–9. doi: 10.1016/j.ejphar.2018.05.003 29750914

[51] Ney LM Wipplinger M Grossmann M Engert N Wegner VD Mosig AS. Short chain fatty acids: key regulators of the local and systemic immune response in inflammatory diseases and infections. Open Biol. (2023) 13:230014. doi: 10.1098/rsob.230014 36977462 PMC10049789

[52] Pérez-Pérez S Domínguez-Mozo MI Alonso-Gómez A Medina S Villarrubia N Fernández-Velasco JI. Acetate correlates with disability and immune response in multiple sclerosis. PeerJ. (2020) 8:e10220. doi: 10.7717/peerj.10220 33240608 PMC7676361

[53] Kujawa D Laczmanski L Budrewicz S Pokryszko-Dragan A Podbielska M. Targeting gut microbiota: new therapeutic opportunities in multiple sclerosis. Gut Microbes. (2023) 15:2274126. doi: 10.1080/19490976.2023.2274126 37979154 PMC10730225

[54] Lobb RR Hemler ME. The pathophysiologic role of alpha 4 integrins *in vivo* . J Clin Invest. (1994) 94:1722–8. doi: 10.1172/JCI117519 PMC294562 7525645

[55] Peled JU Gomes ALC Devlin SM Littmann ER Taur Y Sung AD. Microbiota as predictor of mortality in allogeneic hematopoietic-cell transplantation. N Engl J Med. (2020) 382:822–34. doi: 10.1056/NEJMoa1900623 PMC7534690 32101664

[56] Taur Y Xavier JB Lipuma L Ubeda C Goldberg J Gobourne A. Intestinal domination and the risk of bacteremia in patients undergoing allogeneic hematopoietic stem cell transplantation. Clin Infect Dis. (2012) 55:905–14. doi: 10.1093/cid/cis580 PMC3657523 22718773

[57] Khan N Lindner S Gomes ALC Devlin SM Shah GL Sung AD. Fecal microbiota diversity disruption and clinical outcomes after auto-HCT: a multicenter observational study. Blood. (2021) 137:1527–37. doi: 10.1182/blood.2020006923 PMC7976512 33512409

[58] Masetti R Leardini D Muratore E Fabbrini M D’Amico F Zama D. Gut microbiota diversity before allogeneic hematopoietic stem cell transplantation as a predictor of mortality in children. Blood. (2023) 142:1387–98. doi: 10.1182/blood.2023020026 PMC10651870 37856089

[59] Vaitkute G Panic G Alber DG Faizura-Yeop I Cloutman-Green E Swann J. Linking gastrointestinal microbiota and metabolome dynamics to clinical outcomes in paediatric haematopoietic stem cell transplantation. Microbiome. (2022) 10:89. doi: 10.1186/s40168-022-01270-7 35689247 PMC9185888

[60] Liu C Frank DN Horch M Chau S Ir D Horch EA. Associations between acute gastrointestinal GvHD and the baseline gut microbiota of allogeneic hematopoietic stem cell transplant recipients and donors. Bone Marrow Transpl. (2017) 52:1643–50. doi: 10.1038/bmt.2017.200 28967895

[61] Kim YS Unno T Kim BY Park MS. Sex differences in gut microbiota. World J Men’s Health. (2020) 38:48–60. doi: 10.5534/wjmh.190009 30929328 PMC6920072

[62] Patangia DV Anthony Ryan C Dempsey E Paul Ross R Stanton C. Impact of antibiotics on the human microbiome and consequences for host health. MicrobiologyOpen. (2022) 11:e1260. doi: 10.1002/mbo3.1260 35212478 PMC8756738

[63] Singh RK Chang HW Yan D Lee KM Ucmak D Wong K. Influence of diet on the gut microbiome and implications for human health. J Transl Med. (2017) 15:73. doi: 10.1186/s12967-017-1175-y 28388917 PMC5385025

